# Citric Acid by-Product Fermentation by *Bacillus subtilis* I9: A Promising Path to Sustainable Animal Feed

**DOI:** 10.3390/vetsci11100484

**Published:** 2024-10-08

**Authors:** Sirisak Tanpong, Nalisa Khochamit, Padsakorn Pootthachaya, Wilailak Siripornadulsil, Narirat Unnawong, Anusorn Cherdthong, Bundit Tengjaroenkul, Sawitree Wongtangtintharn

**Affiliations:** 1 Department of Animal Science, Faculty of Agriculture, Khon Kaen University, Khon Kaen 40002, Thailand; sirisakt@kkumail.com (S.T.); padsakornp@kkumail.com (P.P.); nariratunnawong@kkumail.com (N.U.); anusornc@kku.ac.th (A.C.); 2 Department of Microbiology, Faculty of Science, Khon Kaen University, Khon Kaen 40002, Thailand; nalisa_khochamit@kkumail.com (N.K.); swilai@kku.ac.th (W.S.); 3 Department of Veterinary Public Health, Faculty of Veterinary Medicine, Khon Kaen University, Khon Kaen 40002, Thailand; btengjar@kku.ac.th

**Keywords:** sustainability challenge, nutritional enhancement, microbial intervention, amino acid enrichment, structural transformation

## Abstract

**Simple Summary:**

Utilizing food industry by-products for animal feed is challenging due to their low nutrient content. Citric acid by-products have potential as feed, but their high fiber content limits their use. In this study, we used *Bacillus subtilis* I9 to improve citric acid by-product quality. Fermentation reduced fiber, increased protein by 21.89%, and improved amino acid ratios. Structural changes were observed using scanning electron microscopy. Using *B. subtilis* I9 to process citric acid waste enhances its nutritional value, making it a healthier choice for animal consumption and aiding sustainability.

**Abstract:**

Citric acid by-products in animal feed pose a sustainability challenge. *Bacillus* species are commonly used for fermenting and improving the nutritional quality of feedstuffs or by-products. An experiment was conducted to enhance the nutritional value of citric acid by-products through fermentation with *Bacillus subtilis* I9 for animal feed. The experiment was carried out in 500 mL Erlenmeyer flasks with 50 g of substrate and 200 mL of sterile water. Groups were either uninoculated or inoculated with *B. subtilis* I9 at 10^7^ CFU/mL. Incubation occurred at 37 °C with automatic shaking at 150 rpm under aerobic conditions for 0, 24, 48, 72, and 96 h. Inoculation with *B. subtilis* I9 significantly increased *Bacillus* density to 9.3 log CFU/mL at 24 h (*p* < 0.05). CMCase activity gradually increased, reaching a maximum of 9.77 U/mL at 72 h. After 96 h of fermentation with inoculated *B. subtilis* I9, the citric acid by-product exhibited a significant decrease (*p* < 0.05) in crude fiber by 10.86%, hemicellulose by 20.23%, and cellulose by 5.98%, but an increase in crude protein by 21.89%. Gross energy decreased by 4% after inoculation with *B. subtilis* in comparison to the uninoculated control (*p* < 0.05). Additionally, the non-starch polysaccharide (NSP) degradation due to inoculation with *B. subtilis* I9 significantly reduced (*p* < 0.05) NSP by 24.37%, while galactose, glucose, and uronic acid decreased by 22.53%, 32.21%, and 18.11%, respectively. Amino acid profile content increased significantly by more than 12% (*p* < 0.05), including indispensable amino acids such as histidine, isoleucine, lysine, methionine, phenylalanine, tryptophan, and valine and dispensable amino acids like alanine, aspartic acid, glutamic acid, glutamine, glycine, proline, and tyrosine. Furthermore, citric acid by-products inoculated with *B. subtilis* I9 exhibited changes in the cell wall structure under scanning electron microscopy, including fragmentation and cracking. These results suggest that fermenting citric acid by-products with *B. subtilis* I9 effectively reduces dietary fiber content and improves the nutritional characteristics of citric acid by-products for use in animal feed.

## 1. Introduction

Citric acid is a vital organic acid produced by the biological fermentation of starch using *Aspergillus niger*. This fermentation process typically utilizes substrates such as rice, corn, cassava, or cassava pulp. The by-products, often referred to as “spent cassava”, are the remaining residues after the citric acid extraction process [1]. These by-products can contain a significant amount of organic matter, including residual sugars, proteins, and fibers, making them potential feed ingredients for livestock or substrates for further processing. The annual production of citric acid surpasses 1.7 million tons. The global market demand for citric acid is rising at about 5% per year [1,2]. However, citric acid production by fermentation typically results in waste residues amounting to approximately 1.6–2 times the weight of the substrate, leading to environmental pollution, and citric acid production by-products, as waste, account for 70.0 to 80% of the total production [3]. There is an urgent need to adopt environmentally friendly citric acid production and discover ways to convert these by-product residues into animal feed, a highly sought-after resource for animal nutrition [4]. Incorporating food industry by-products into animal feed poses a sustainable management challenge due to their low nutrient content and limited animal digestibility. Citric acid by-products contain cellulose, hemicellulose, sugars, starch, and protein. For instance, Tanpong et al. [5] reported that the chemical composition of citric acid by-products contained 6.11% crude protein (CP), 2.39% ether extract (EE), 18.26% nitrogen-free extract (NFE), and 52.73% crude fiber (CF) and had a gross energy (GE) of 3.59 Mcal/kg, making them a valuable energy source for animals. Oryza et al. [6] also reported the chemical composition of citric acid by-products from rice, which included 19.80% CP, 3.98% EE, 46.64% NFE, 11.97% CF, and a GE of 4.00 Mcal/kg. One notable advantage of these by-products is their affordability, which can help reduce feed costs. However, Tanpong et al. [7] found that using citric acid by-products at 9% in quail diets can negatively impact growth performance due to their high fiber content, which affects nutrient digestibility and growth. There have been attempts to use microbial fermentation to deal with the dietary fiber component. In the process of metabolism, microbes may release specific enzymes that reduce the molecular weight of lignin, cellulose, and hemicellulose [8]. *Bacillus*, a well-known cellulose-degrading microorganism, has been utilized to enhance the nutritive value of various substrates, including soybean, fruit by-products, corn ethanol by-products, and cassava [9,10].

*Bacillus subtilis* has shown that it can make cellulolytic enzymes, such as cellobiase-rich cellulase and endo-glucanase, using agricultural waste as its only carbon source [11]. *Bacillus* spp. can ferment substrates to release hydrolytic enzymes that help break down and use oligosaccharides, polysaccharides, and antinutrients that the body cannot digest. These enzymes also help break down proteins, amino acids, and phytochemicals, making the substrate more suitable for animal nutrition [12].

In recent years, microorganisms have played a crucial role in enhancing the nutritional quality of feed ingredients and by-products used in animal nutrition through fermentation. This study hypothesizes that *B. subtilis* fermentation will improve citric acid by-products’ nutritional quality and chemical composition, making them more useful for animal feed. This study aims to use *B. subtilis* fermentation to add value to citric acid by-products from the industry and improve their chemical composition characteristics, making them a viable option for animal feed.

## 2. Materials and Methods

### 2.1. Sampling of Citric Acid By-Product

The study was conducted in a laboratory at the Department of Animal Science, Faculty of Agriculture, and the Department of Microbiology, Faculty of Science, Khon Kaen University, Thailand. The samples were collected through surveys, and sampling was used to collect by-products from citric acid producers in the eastern region of Thailand. The starting material consisted of citric acid by-products, derived from cassava. Citric acid was extracted from cassava root through a fermentation process utilizing *Aspergillus niger*. The remaining residues after citric acid extraction constituted the citric acid by-products. The total weight of each sample (5 kg) was collected by random sampling of ten bags (50 kg/bag) using a tapered bag trier. The samples were carefully handled to maintain their original integrity and preserve the nutrient contents of the by-products before conducting fermentation. Prior to use, the citric acid by-products were stored in plastic bags at a controlled temperature of 25 °C to preserve their integrity and prevent spoilage.

### 2.2. Microorganisms

#### 2.2.1. Bacterial Strain

The *Bacillus* strain was isolated from the small intestine of the broiler in a laboratory at the Faculty of Science, Khon Kean University, Thailand. *Bacillus* strain was confirmed for *B. subtilis* isolation 9 (*B. subtilis* I9) by gram stain, enzyme activity, and biochemical tests.

#### 2.2.2. 16S rDNA Sequencing and Data Analysis

To confirm *B. subtilis* I9 by molecular sequencing technique, *B. subtilis* I9 was cross streak onto Luria–Bertani (LB) medium (HiMedia Laboratories Pvt, Ltd., Mumbai, India). The LB plates were incubated at 37 °C for 36–48 h and re-streaked on LB plates again. A single colony was transferred into LB broth for DNA extraction and polymerase chain reaction (PCR) using 27F/1492R universal primers, with a product size of 1496 bp. Then 785F and 907R primers were used for sequencing as forward and reverse sequences, respectively. *B. subtilis* was confirmed with 16S rRNA sequencing. The primers used for PCR amplification were 27F 5′ (AGA GTT TGA TCM TGG CTC AG) 3′ and 1492R 5′ (TAC GGY TAC CTT GTT ACG ACT T) 3′. The primers used for sequencing were 785F 5′ (GGA TTA GAT ACC CTG GTA) 3 and 907R 5′ (CCG TCA ATT CMT TTR AGT TT) 3′ for forward and reverse sequences, respectively. The computer analysis of the 16S rRNA sequences was performed by comparing them with sequences in GenBank.

### 2.3. Preparation of Inoculated Mixed By-Product

We grew *B. subtilis* I9 on Luria Bertani (LB) plates, which were previously sterilized in LB broth at 37 °C overnight with shaking (150 rpm) for 24 h. Ten replicate plates were prepared for each. We diluted the cultures at a ratio of 1:10 in LB and allowed them to grow at 37 °C under agitation (150 rpm), regularly measuring the optical density (OD) at 600 nm. The total bacteria count was represented by colony-forming units (CFU), determined through serial dilution using a drop plate method at 0, 3, 6, 9, 12, 18, 24, 36, 48, and 72 h of incubation.

*B. subtilis* I9 was cultured and incubated in LB broth medium at 37 °C for 24 h before fermentation. Before fermentation, the by-product substrates were sterilized to reduce the effects of exogenous microorganisms during the fermentation process. Each substrate contained 50 g and was combined with 200 mL of sterile water in a 500 mL Erlenmeyer flask, which was covered with cotton plugs. The groups consisted of either uninoculated or inoculated samples (*B. subtilis* I9 at 10^7^ CFU/mL) incubated at 37 °C in an auto-shaking incubator (150 rpm) under aerobic conditions. Fermentation times were set at 0, 24, 48, 72, and 96 h, with five replications for each fermentation time. The decision to use an initial density of 7 log CFU/mL for *Bacillus subtilis* I9 was based on recommendations from previous studies, which have demonstrated that this concentration is particularly effective in various applications [11,12].

Samples were collected for analyses, such as reducing sugar, enzyme assay, and bacterial count. After incubation, wet samples were collected and treated at 105 °C for 30 min to stop fermentation. The samples were dried in a hot air oven at 60 °C for 48 h to analyze chemical compositions, amino acid profiles, and non-starch polysaccharides of by-products. Prior to use, the citric acid by-products were stored in plastic bags at a controlled temperature of 25 °C to preserve their integrity and prevent spoilage.

### 2.4. Enzyme Assay

After incubation, the collected samples were inoculated and centrifuged at 15,000× *g* for 10 min to obtain the supernatant for the enzyme assay. The enzyme activity assay, as described by Malik et al. [13], used 1% carboxymethyl cellulase (CMCase) in 0.05 M sodium citrate buffer (pH 4.8), which was incubated for 30 min at 50 °C. The reducing sugar concentration was measured using the dinitrosalicylic acid (DNS) described by Chen et al. [14]. One unit (U) of enzyme was defined as the amount of enzyme that produces 1 µmol of glucose per minute under assay conditions.

### 2.5. Chemical Compositions

The samples were carefully handled to maintain their original integrity for study on chemical properties in the Laboratory of the Department of Animal Science, Faculty of Agriculture, Khon Kaen University, Khon Kaen, Thailand. The dried samples of citric acid by-product, either uninoculated or inoculated (96 h), were analyzed for chemical composition via proximate analysis, including moisture, ash, crude protein (CP), crude fiber (CF), ether extract (EE), nitrogen-free extract (NFE), calcium (Ca), and phosphorus (P), as determined using the methods of AOAC [15]. Gross energy (GE) was measured using adiabatic bomb calorimeters (AC 500, Leco, Ltd., St. Joseph, MI, USA). Neutral detergent fiber (NDF) and acid detergent fiber (ADF) were assessed following the methodology outlined by Van Soest et al. [16], employing the filter bag technique (Ankom Technology, Macedon, NY, USA). Acid detergent lignin (ADL) analysis was conducted in accordance with the procedures described by Raffrenato and Van Amburgh [17]. Hemicellulose content was calculated as the difference between NDF and ADF, and cellulose content was calculated as the difference between ADF and ADL.

Non-starch polysaccharide (NSP) components were measured on the basis of alditol acetates, as determined by gas-liquid chromatography for monosaccharides, and uronic acid was measured by a colorimetric method based on the procedure described by Toucheteau et al. [18], where 2M sulfuric acid was used for the hydrolysis of non-cellulosic polysaccharides.

The amino acid profile was extracted following the method described by Nimbalkar et al. [19]. Amino acid analysis was conducted following the method outlined by Onozato et al. [20], employing liquid chromatography with tandem mass spectrometry (LC-MS/MS) systems. LC-MS/MS analysis was conducted using a triple quadrupole tandem mass spectrometer (Shimadzu Corp., Kyoto, Japan) coupled with a 1290 infinity LC system (Agilent Technologies, Santa Clara, CA, USA). Chromatographic separation of amino acids was performed on an Atlantis Silica HILIC column measuring 4.6 mm × 100 mm, with a particle size of 3 m (Waters Corporation, Midford, MA, USA).

### 2.6. Scanning Electron Microscopy (SEM)

A scanning electron microscope (JEOL-JSM 6460 LV, Tokyo, Japan) was used to examine the shape of the fermented citric acid by-product. A 3 g sample was mounted on the stub with double-sided adhesive tape carbon and sputter coated with a gold layer (40–50 nm) under high vacuum mode. Ultrastructure analysis of the samples was carried out using a scanning electron microscope at an accelerating voltage of 20 kV, following the method reported by Tanpong et al. [5] and Oryza et al. [6]. Images were captured from three replicates for each sample in the series.

### 2.7. Statistical Analysis

The data were analyzed using one-way analysis with the general linear models in the SAS procedure [21]. Differences among means with a *p*-value of less than 0.05 were considered significant.

## 3. Results

### 3.1. 16S rDNA Sequencing

**Figure 1:**
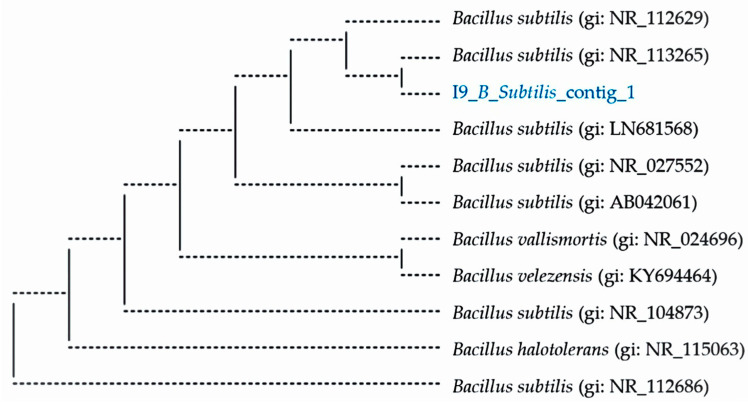
Phylogenetic tree analysis based on partial 16S rDNA sequences of *B. subtilis* I9.

*B. subtilis* I9 sequencing is shown in Figure 1 as (I9_*B_Subtilis*_coting_1). Sequences producing significant alignments with a length of 1496 bp matched those of *B. subtilis*, such as > CP020102.1 *B. subtilis* strain NCIB 3610 chromosome, complete genome, length = 4215607, score = 2739 bits (1483), expect = 0.0, identities = 1483/1483 (100%), Gaps = 0/1483 (0%), strand = plus/plus. Computer analysis of the 16S rRNA sequences was performed by comparison with sequences in the GenBank of *B. subtilis* according to biochemical results. DNA sequencing result was 1496 bps. The DNA sequencing results indicated that *Bacillus* I9 was scientifically classified as Kingdom: Bacteria, Family: Bacillaceae, Genus: *Bacillus*, Species: *B. subtilis*, as shown in Figure 1.

**Figure 2:**
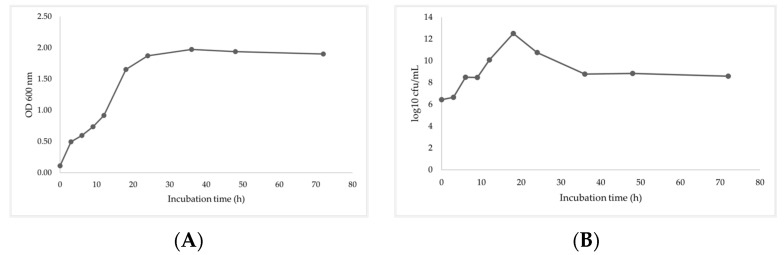
Growth curve of *B. subtilis* I9 on (**A**) OD 600 and (**B**) log CFU/mL.

*B*. *subtilis* I9 colonies grown on LB media exhibited an optical density at 600 nm (OD 600) and bacterial density (log CFU/mL). The lag phase of bacteria occurred within 3 h, and the log phase occurred after 12 h of incubation. *B. subtilis* I9 reached its maximum growth at 18 h. It then declined into the death phase, as shown in Figure 2A,B.

### 3.2. B. subtilis Density and Enzyme Assay during Fermentation

**Figure 3:**
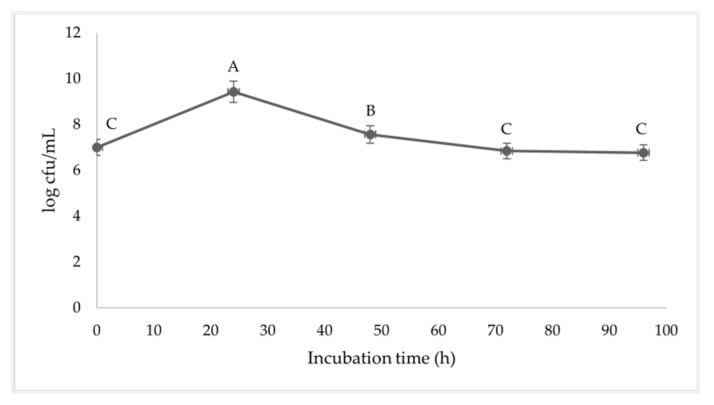
Growth curve of *B. Subtilis* during inoculation after 96 h (log CFU/mL). ^A–C^ Means within rows with different superscript letters differ at *p* < 0.05.

The effect of incubation of *B*. *subtilis* I9 growth is shown in Figure 3, where the fermentation was prolonged and inoculated with a by-product substrate. While specific time points like 0, 24, 48, 72, and 96 h have their appeal, our approach of using a continuous timeline offers several advantages, such as more accurately capturing growth curves, enzyme activity trends, and subtle variations, ultimately providing a clearer and more comprehensive understanding of the dynamic biological processes being studied. The initial density of the *B. subtilis* incubation was 7 log CFU/mL. After 24 h of inoculation, there was a maximum proliferation of 9.3 log CFU/mL (*p* < 0.05). The density of *B. subtilis* gradually decreased to 7.4, 7.0, and 6.6 log CFU/mL after 48, 72, and 96 h of inoculation.

**Figure 4:**
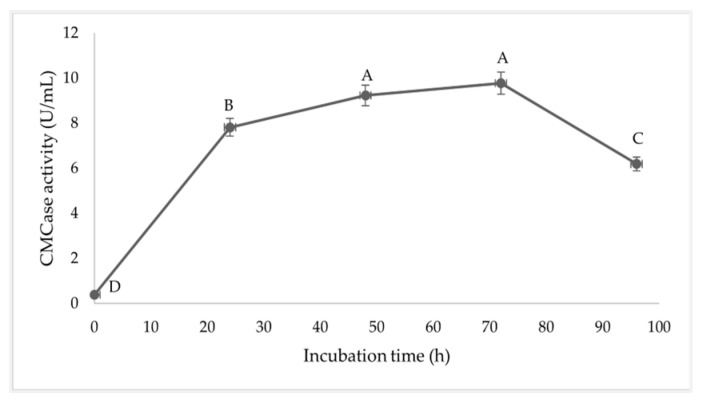
Enzyme CMCase activity during inoculation after 96 h. ^A–D^ Means within rows with different superscript letters differ at *p* < 0.05.

Enzyme CMCase activity during inoculation is shown in Figure 4. The CMCase activity at the initial incubation was 0.39 U/mL. It increased to 7.82 and 9.23 U/mL after 24 and 48 h, which reached a maximum level of 9.776 U/mL after 72 h of incubation (*p* < 0.05). However, the CMCase activity gradually decreased to 6.19 U/mL after 96 h of inoculation.

### 3.3. Chemical Compositions

The analyzed chemical compositions of fermentation, both inoculated and uninoculated with by-products after 96 h of incubation, are presented in Table 1. The results showed that the inoculated by-product significantly (*p* < 0.05) increased CP by 21.89% and decreased CF by 10.86%, while gross energy was reduced by 4% compared with the uninoculated. NDF, ADF, hemicellulose, and cellulose of inoculated by-products showed significant (*p* < 0.05) decreases by 8.62, 4.47, 20.23, and 5.98%, respectively, compared with the uninoculated.

The NSP compositions of the inoculated citric acid by-product are presented in Table 2. The inoculation of *B. subtilis* I9 significantly (*p* < 0.05) degraded total NSP by 24.37%, whereas galactose, glucose, and uronic acid degraded by 22.53, 32.21, and 18.11%, respectively, compared with uninoculated.

The amino acid profiles of fermentation, both inoculated and uninoculated, are presented in Table 3. The inoculation significantly (*p* < 0.05) improved the indispensable amino acids, including His, Ile, Lys, Met, Phe, Trp, and Val, and dispensable amino acids, including Ala, Asp, Glu, Gln, Gly, Pro, and Tyr, compared with the uninoculated control. Notably, these amino acids increased by more than 12% in the by-products.

### 3.4. Scanning Electron Microscopy

**Figure 5:**
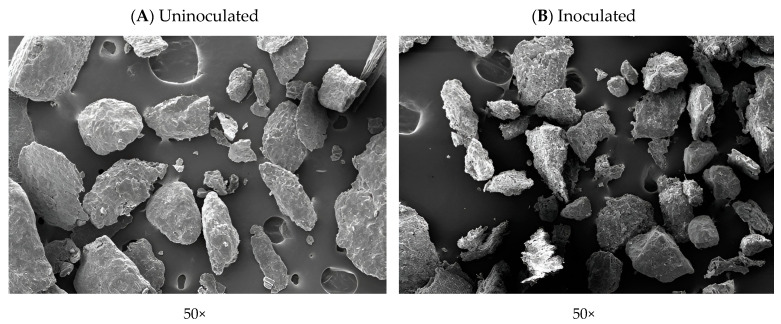
Scanning electron microscopy of the cell wall of citric acid by-product with samples uninoculated (**A**) and inoculated with *B*. *subtilis* I9 (**B**), after fermentation under aerobic conditions at 37 °C for 96 h. All the images were taken at 50× magnification.

Microscopic images of the cell wall of the citric acid by-product after being uninoculated and inoculated were obtained by scanning electron microscopy and are presented in Figure 5. The ultrastructure morphology of the uninoculated sample’s observed surface of the cell wall is smooth, whereas the inoculated sample with *B. Subtilis* I9 showed the cell wall structure to be fragmental and cracked under ×50, ×500, and ×1000 magnifications.

## 4. Discussion

The process of citric acid production normally generates by-product residues, which cause environmental pollution. A major advantage of by-products is reduced feed costs when used as feed ingredients. However, by-products contain high fiber levels that limit the utilization of monogastric animal diets [5]. Several reports have shown negative effects of fiber content on the digestibility of proteins and lipids. Current studies have investigated whether microbial fermentation can reduce dietary fiber, degrade anti-nutritional properties, and improve nutritional characteristics. *B. subtilis* is a naturally occurring endospore-forming bacterium. Its spores can resist highly extreme environments. Its advantage is that it can be used in industry and the application of probiotics to improve immune function, inhibit pathogenic bacteria, maintain health, and improve nutritive value [22]. In this experiment, *B. subtilis* was confirmed with 16S rRNA sequencing before being inoculated with by-products for fermentation processing, which improved nutritional quality. *B. subtilis* I9 growth in LB was slow during the first 3 h of incubation and increased to 6.66 log CFU/mL. Then, *B. subtilis* exponentially increased during the 9–18 h and reached a maximum of 12.51 log CFU/mL at incubation.

At the end of the log phase at 18 h, the duration of the stationary phase was 36 h of the incubation time. However, after 48 h, the density of the bacteria slightly decreased with decreasing nutrient concentration. The fermentation by-product with *B. subtilis* I9 was inoculated during the fermentation process. The fermentation of the by-product with *B. subtilis* I9 that was inoculated during 24 h was exponentially increased and reached its maximum, and after 24–72 h, it slightly declined until it stablized. The reducing sugar concentration decreased rapidly during the exponential growth phase (0–24 h). However, the reducing sugar did not decrease after 72 h and remained at 4 mg/mL until the end of fermentation. *B. subtilis* demonstrated that the metabolite produced was enzyme CMCase productivity. The enzyme product was exponentially increased for 24 h, reached a maximum at 48–72 h (9.77 U/mL), and gradually declined by 96 h. Wang et al. [23] reported that the CMCase activity produced by *B. paralicheniformis* Y4 rapidly increased up to 72 h with a maximum CMCase activity of 8.96 U/mL. Jiménez-Leyva et al. [24] reported that *B. subtilis* RZ164, RS351, and RS273 isolated from corn stover produced CMCase activity that was detected after 24 h. This activity rapidly increased, reaching its highest levels at 48–72 h of incubation. Bacteria growth using carbon substrates has a dual role in biosynthesis and energy generation. The use of carbohydrates as a carbon source for microbial fermentation processes led to an increase in bacterial cell density. Additionally, this resulted in the production of metabolites and enzymes, as well as a decline in reducing sugars [25].

In this work, by-product fermentation with inoculated products contained greater crude protein and amino acid compositions when compared with uninoculated products, which showed significant differences. The inoculated *B. subtilis* I9 levels increased crude protein (21.89%) and total amino acid (108.04%) compared to the uninoculated samples. The amino acid profiles showed a difference between the inoculated and uninoculated groups. Our results aligned with Shi et al. [26], who observed a significant difference in the increased CP of 14.80% and total amino acid of 7.90% after fermentation by *B. subtilis* and *E. faecium*. Likewise, Chen et al. [27] reported that the fermentation by *B. velezensis* and *L. plantarum* improved nutrient composition by increasing CP by 9.90% and total amino acid (AA) by 12.10%. The reason for increased CP could be the loss of dry matter, mainly carbohydrates, during fermentation, which led to an increase in the concentration of nutrients. This is supported by Suriyapha et al. [2], who explained that dry matter loss during fermentation led to increased protein and amino acid content. However, the protein and amino acid profiles of *B. subtilis* I9 contained protein (46.50%) and amino acids (mostly alanine, leucine, and glutamic acid). Our results were in line with the findings of Sarabandi et al. [28], who reported that *B. cereus* contained protein, and the amino acids with the highest content were aspartic acid, glutamic acid, alanine, glycine, leucine, and threonine. Likewise, Munoz and Sadaie [29] revealed that the *B. subtilis* spore coat protein accounts for approximately 10% of the total dry weight of spores and 25% of the total protein content of *B. subtilis* spores. Therefore, the inoculated feed with *B. subtilis* can increase protein and amino acid content more than the uninoculated feed. The inoculated by-product’s improved nutritional composition, including increased essential amino acids and reduced anti-nutritional factors, might significantly benefit animals’ health [11]. This balanced amino acid profile is crucial for growth, tissue development, and immune function, enhancing nutrient utilization and performance [12].

Nevertheless, the current study demonstrated that the citric acid by-product fermented with *B. subtilis* exhibited reduced gross energy compared to the uninoculated sample. This may be attributed to *B. subtilis* fermentation, which may cause the degradation of certain nutrients, including carbohydrates, leading to a decrease in gross energy. The energy contained in these nutrients is lost during fermentation, which may lead to a loss of energy upon feed consumption [2]. Therefore, the subsequent use of citric acid by-product incubated with *B. subtilis* in animal feed must provide a balanced energy value to prevent suboptimal animal performance.

In the experiment, we observed that the chemical composition was altered during the fermentation with *B. subtilis* I9; crude fiber, hemicellulose, and cellulose levels showed significant declines of 10.86, 20.23, and 5.98%, respectively. Moreover, the fermentation showed degradation of the NSP, such as total NSP (24.37%), galactose (22.59%), glucose (32.21%), and uronic acid (18.14%), which were most significantly altered during fermentation with by-product inoculated with *B. subtilis* I9 compared with uninoculated. Consequently, the fermentation observed in the structural analysis of the by-product by microscopy of SEM showed cracks, small fractions, and porousness in the cell wall during the altered incubation. This demonstrated the breakdown of the cell wall of the by-product by enzymes produced by *B. subtilis* I9. The lower levels of crude fiber, cellulose, hemicellulose, and non-starch polysaccharides (NSP) indicated that *B. subtilis* I9 would produce enzymes that hydrolyze cellulose, hemicellulose, and NSP during the fermentation process. This could improve nutrient composition, crude protein, amino acids, and digestibility compared to the uninoculated by-product. Our results were in alignment with Shi et al. [26], who reported that two-stage fermentation using *B. subtilis* and *E. faecium* showed a degraded anti-nutritional factor and decreased cellulose and hemicellulose. This process enhanced levels of protein and amino acids in soybean meal, improving the digestibility of crude protein and amino acids more than in unfermented samples. Likewise, studies by Chen et al. [27] reported that *B. velezensis* and *L. plantarum* significantly declined cellulose and hemicellulose in soybean meal. Many enzymes have been produced by *B. subtilis*, for instance, amylases, xylanases, lichenases, β-galactosidases [28,29], cellulases [30,31], proteases [32], and many others. The cellulase group of enzymes is made up of multiple enzymes, such as endoglucanases, exoglucanases, and glycosidases, which act together to hydrolyze the cellulose molecule. Endoglucanases are widely used and comprise the endo1,4-β glucanase enzyme that hydrolyzes the cellulose chains at random [33]. Endoglucanases, which are mostly carboxymethyl cellulases (CMCases), can release small fragments of cellulose with both reducing and non-reducing ends. The next step involves exoglucanases releasing cellobiose and short oligosaccharides [34]. Hemicellulose is hydrolyzed by the enzymes 1,4-β-xylosidase, endo-1,4-β-xylanase, arabinan endo-1,5-α-l-arabinosidase, α-N-arabinofuranosidase, ara-binoxylan arabinofuranohydrolase, β-mannosidase, arabinogalactan endo-1,4-β-galactosidase, and glucuronoxylanase. Therefore, *B. subtilis* I9 is used in fermentation to enhance the nutrient composition of the by-product of citric acid, decrease fiber, cellulose, and hemicellulose, and improve crude protein and amino acid profiles for use as feed ingredients [35].

## 5. Conclusions

Our findings demonstrate the potential of citric acid by-product fermentation using *B. subtilis* I9 as a promising strategy for enhancing the nutritional value of agricultural by-products. *B. subtilis* I9, equipped with CMCase activity, effectively hydrolyzed NSP, cellulose, and hemicellulose, leading to improved protein and amino acid composition during fermentation. This balanced amino acid profile may improve the growth of animals, immunological functions, and nutrition utilization. These results highlight the potential of *B. subtilis* I9 to enhance the nutritional quality of by-products, making them more suitable for use as animal feed. The present work underscores the need for further research to validate and expand upon our preliminary findings. Specifically, future studies should focus on the long-term impact of CABR supplementation on the zootechnical characteristics of broiler chickens, including meat and fat quality. The study revealed that citric acid by-product fermented with *B. subtilis* may reduce gross energy due to nutrient degradation, highlighting the need for balanced energy values in animal feed. Additionally, optimizing the fermentation process for maximum nutritional benefits and evaluating the specific effects of fermented by-products on animal growth performance are critical steps for advancing this research.

## Figures and Tables

**Table 1:** Chemical composition of citric acid by-product fermentation with uninoculated and inoculated samples.

Chemical Compositions (%DM)	Uninoculated	Inoculated	SEM	*p*-Value
Dry matter	93.28	93.15	0.26	0.73
Ash	13.70	13.88	0.36	0.73
Crude protein	7.39 ^b^	9.01 ^a^	0.26	<0.01
Ether extract	2.97	3.06	0.13	0.63
Crude fiber	18.26 ^a^	16.28 ^b^	0.09	<0.01
Nitrogen free extract	50.96	50.92	0.49	0.95
Calcium	1.03	1.08	0.09	0.69
Phosphorus	0.10	0.12	0.01	0.21
Gross energy (kcal/kg)	3819.00 ^a^	3666.00 ^b^	17.60	<0.01
Neutral detergent fiber	59.75 ^a^	54.60 ^b^	0.93	0.02
Acid detergent fiber	44.05 ^a^	42.08 ^b^	0.42	0.03
Acid detergent lignin	14.92	14.69	0.19	0.45
Hemicellulose	15.71 ^a^	12.53 ^b^	0.59	0.02
Cellulose	29.13 ^a^	27.39 ^b^	0.38	0.03

^a,b^ Means within rows with different superscript letters differ at *p* < 0.05.; SEM: standard error of mean.

**Table 2:** NSP composition of citric acid by-product fermentation with uninoculated and inoculated samples.

Parameters	Uninoculated	Inoculated	Degradation (%)	SEM	*p*-Value
NSP	25.83 ^a^	19.54 ^b^	24.37	0.08	<0.01
Arabinose	1.08	0.92	15.32	0.05	0.09
Xylose	2.13	1.93	9.70	0.07	0.09
Mannose	0.49	0.43	11.60	0.02	0.07
Galactose	2.32 ^a^	1.79 ^b^	22.53	0.09	0.02
Glucose	12.44 ^a^	8.43 ^b^	32.21	0.13	<0.01
Uronic acid	1.65 ^a^	1.38 ^b^	18.11	0.15	<0.01

^a,b^ Means within rows with different superscript letters differ at *p* < 0.05.; SEM: standard error of mean.

**Table 3:** Amino acid (AA) profiles of citric acid by-product fermentation with uninoculated and inoculated samples.

AA Compositions (%)	*B. Subtilis* I9	Uninoculated	Inoculated	SEM	*p*-Value
Indispensable amino acid					
Arginine (Arg)	0.290	0.213	0.194	0.012	0.305
Histidine (His)	1.260	0.060 ^b^	0.067 ^a^	0.058	<0.001
Isoleucine (Ile)	4.220	0.065 ^b^	0.348 ^a^	0.025	0.001
Leucine (Leu)	5.090	0.198 ^b^	0.326 ^a^	0.030	0.042
Lysine (Lys)	2.640	0.028 ^b^	0.190 ^a^	0.005	<0.001
Methionine (Met)	1.680	0.008 ^b^	0.034 ^a^	0.002	0.002
Phenylalanine (Phe)	3.900	0.054 ^b^	0.235 ^a^	0.011	<0.001
Threonine (Thr)	0.530	0.094	0.069	0.012	0.192
Tryptophan (Trp)	0.600	0.012 ^b^	0.086 ^a^	0.003	<0.001
Valine (Val)	2.950	0.062 ^b^	0.159 ^a^	0.007	0.001
Dispensable amino acid					
Alanine (Ala)	5.530	0.167 ^b^	0.683 ^a^	0.017	<0.001
Aspartic acid (Asp)	0.190	0.037 ^b^	0.074 ^a^	0.003	0.002
Asparagine (Asn)	0.170	0.021	0.016	0.006	0.886
Cystein (Cys)	0.130	0.006	0.006	0.000	0.195
Glutamic acid (Glu)	7.330	0.146 ^b^	0.235 ^a^	0.015	0.017
Glutamine (Gln)	2.640	0.057 ^b^	0.118 ^a^	0.006	0.003
Glycine (Gly)	0.640	0.043 ^b^	0.059 ^a^	0.002	0.008
Proline (Pro)	2.860	0.245 ^b^	0.608 ^a^	0.021	<0.001
Serine (Ser)	3.560	0.001	0.001	0.001	0.218
Tyrosine (Tyr)	0.290	0.470 ^b^	0.710 ^a^	0.045	0.018
Total AA	46.500	1.990 ^b^	4.140 ^a^	0.032	<0.001

^a,b^ Means within rows with different superscript letters differ at *p* < 0.05.; SEM: standard error of mean.

## Data Availability

The data presented in this study are available upon request from the corresponding author.
